# Openness to Change: Experiential and Demographic Components of Change in Local Health Department Leaders

**DOI:** 10.3389/fpubh.2015.00209

**Published:** 2015-09-01

**Authors:** Emmanuel D. Jadhav, James W. Holsinger, David W. Fardo

**Affiliations:** ^1^Public Health Programs, Ferris State University, Big Rapids, MI, USA; ^2^Department of Preventive Medicine, University of Kentucky, Lexington, KY, USA; ^3^Department of Biostatistics, University of Kentucky, Lexington, KY, USA

**Keywords:** public health leadership, organization theory, openness to change, public health management, workforce development

## Abstract

**Background:**

During the 2008–2010 economic recession, Kentucky local health department (LHD) leaders utilized innovative strategies to maintain their programs. A characteristic of innovative strategy is leader openness to change. Leader demographical research in for-profit organizations has yielded valuable insight into leader openness to change. For LHD leaders, the nature of the association between leader demographic and organizational characteristics on leader openness to change is unknown. The objectives of this study are to identify variation in openness to change by leaders’ demographic and organizational characteristics and to characterize the underlying relationships.

**Materials and Methods:**

The study utilized Spearman rank correlations test to determine relationships between leader openness to change (ACQ) and leader and LHD characteristics. To identify differences in the distribution of ACQ scores, Wilcoxon–Mann–Whitney and Kruskal–Wallis non-parametric tests were used, and to adjust for potential confounding, linear regression analysis was performed.

**Data:**

Local health department leaders in the Commonwealth of Kentucky were the unit of analysis. Expenditure and revenue data were available from the state health department. National census data were utilized for county level population estimates. A cross-sectional survey was performed of KY LHD leaders’ observable attributes relating to age, gender, race, educational background, leadership experience, and openness to change.

**Results:**

Leaders had relatively high openness to change scores. Spearman correlations between leader ACQ and departmental 2012–2013 revenue and expenditures were statistically significant, as were the differences observed in ACQ by gender and the educational level of the leader. Differences in ACQ score by education level and agency revenue were significant even after adjusting for potential confounders. The analyses imply that there are underlying relationships between leader and LHD characteristics based on leader openness to change.

## Introduction

Local health department (LHD) leaders used innovative strategies to withstand external financial pressures linked to internal quality challenges and service volume during the 2008–2010 economic recession (1). The strategies included charging fees for services and billing insurance, pursuing new funding sources, hiring contractors, utilizing technology, as well as contracting for and/or sharing staff or equipment with other LHDs or non-LHD organizations (2). While several leader characteristics influence the organization’s performance, central to the organization’s ability to innovate strategically is the leader’s openness to change, based on the leader’s ability to examine the rationale and need for specific changes (3). Research in organizational and demographic characteristics of for-profit organization leaders have provided valuable insights into the behavior of top executives and their ability to anticipate issues and take action (4). Little is known about the nature of the relationship between openness to change and organizational and demographic characteristics in LHD leaders. This is among the first studies to identify and characterize variations and underlying relationships in LHD leader openness to change.

In public health agencies, leaders’ openness to change is of particular interest as LHD leaders are consistently faced with surprises and emergencies (5). In addition to managing the historic cycles of action and inaction associated with public health funding (6), leaders’ openness to change is also integral to their ability to control and direct LHDs in a disruptive environment (7). The public expectation that LHD leaders would actively identify financial resources to deliver public health programs (8) during the economic recession is an example of the difficult task that leaders face in finding solutions to problems that are without ready solutions (9).

## Background

Studies have identified that in environments of high uncertainty, leaders exert the most influence on change (10). Indifference toward learning from the environment or competitors leads to poor agency performance (11), implying that a leader’s openness to change is central to the adaption process, suggesting that leaders determine their organizations’ response to a threat or opportunity outside their organizational environment (12) by moving them toward a future state (13). Several organizational and management theory studies have documented the leader’s influence on change management (14, 15) and agency performance (16, 17) and the results are mixed concerning the role of the leader in managing change and agency outcomes (18–20). According to organizational theory, a determinant of openness to change is the perception of the leaders (21), which is driven by the leaders’ attitudes and perceived control over their ability to implement change. This line of inquiry is not without its critics, who suggest that relying on leader demographic characteristics alone (22, 23) without studying their psychological values and attitudes may lead to spurious conclusions (24). These contrasting perspectives on the importance (25) and non-importance (26) of the individual leader’s attributes suggest that although there is no consensus on how demographic attributes drive a leader’s openness to change, the individual leader’s openness to change is important in managing change.

For public health agencies, the 2002 IOM report on educating public health professionals for the twenty first century in response to the 1988 IOM report’s call for the development of the public health workforce in terms of practice and leadership (27) indicated that communities most successful in producing desired health and social outcomes tend to have significant leadership capacity among other agency and community attributes (28). The IOM report, “Variation in Health Care Spending: Target Decision Making, Not Geography” identified that variation over time in utilization of health care services is attributable to decision making that occurs at the level of the each organization (29). In another study, Keane (30) determined that LHD leaders played a significant role as influential decision makers with regard to the privatization of LHD clinical services. These studies suggest that LHD workforce development will benefit from understanding the relationship between the individual leader’s demographic characteristics and role in change management.

## Theoretical Framework

Change management theories based on agency performance criterion have been studied in multiple organizational structures (31). Theories, including the Theory of Planned Behavior, the Upper Echelon Theory, and the Flexible Leadership Theory, provide the basis for understanding the relationship between characteristics of leaders and LHDs and the individual leader’s openness to change.

### Theory of planned behavior

According to this theory, intention to act is antecedent to behavior. The intention to act is a function of the individual’s attitude toward the behavior, subjective norm, and perceived behavioral control. The subjective norm is the individual’s belief concerning whether specific people approve or disapprove of a planned behavior, which is the motivator for the individual to behave such as to gain the group’s approval (32). Perceived behavioral control is the control an individual exercises when performing a behavior (32). These determinants of intention shape the individual’s beliefs about the likely consequences of a specific behavior, expectations of its importance to others, and factors that control behavioral performance. The theory provides a plausible explanation for the interaction between individual attitudes and behaviors (33).

### Upper echelon theory

Founded on the concept of bounded rationality, this theory suggests that complex information and uncertain situations are not objectively known but merely interpreted through the leader’s actions (34). The actions of leaders are in turn guided by their personal interpretation of the situation, and by their personalized construals, values, and personality. Personalized construal in social psychology refers to individual perceptions, comprehension, and interpretation of the world (35) and is a function of experience. The theory suggests that the leaders’ demographic characteristics shape the perceptions of leaders and thereby their openness to change. The theory also accounts for managerial discretion, which is latitude of action in the absence of constraints (36). The concept of managerial discretion explains why some leaders may be more open to change than the others (37).

### Flexible leadership theory

Conceptualized at the organizational level, the four components of this theory are organizational effectiveness, performance determinants, situational variables, and leadership decisions and actions (38). According to the theory, organizational effectiveness is determined by: (a) efficiency and process reliability, (b) human capital, and (c) adaptation to the external environment. The theory accounts for the agency attributes of organization, capacity, and function in shaping the leader’s perception toward change.

## Materials and Methods

The units of analysis were the LHD leaders in the Commonwealth of Kentucky. The 59 LHDs of Kentucky assist in providing a likely comparison to the socioeconomic variations observed in LHDs across the United States during the 2008–2010 economic recession. A cross-sectional cohort study design was used to collect data on openness to change and leader demographics and LHD attributes. This study received an exemption from the University of Kentucky’s institutional review board based on utilizing data that did not identify individual subjects or put individuals at risk.

### Data sources and measures

In 2012, primary data were collected on observable demographic attributes of LHD leaders, such as age, gender, race, educational background, leadership experience, and openness toward change. Of the 59 LHDs in Kentucky, responses were received from 47 leaders resulting in a response rate of nearly 80%. The county level population estimates were available from the US Census Bureau annual county population estimates. The 2012–2013 revenues and expenditures for each LHD were obtained from the Kentucky Department of Public Health. To detect and address anomalies in revenues and expenditures and county level population changes, exploratory analyses and descriptive statistics were performed. The responses were compared internally and with existing data to ensure accuracy.

#### Primary Variable

The openness to change score was measured by Hage and Dewar’s instrument (39), which was developed to specifically measure openness to change in leaders of non-profit organizations. The instrument defines openness to change as the degree to which respondents view change favorably and are therefore more inclined to produce change in their organizations. The scale consists of five items, requiring participants to rate the extent to which they agree with each item on a five-point scale ranging from strongly disagree (1) to strongly agree (5). The openness to change score is the sum of rated responses and reflects the self-assessed openness to change score of the respondent. Lower scores reflect a conservative or non-change-seeking attitude whereas high scores represent a more liberal or change-seeking attitude. The Cronbach’s alpha test was used to measure the instrument’s internal consistency.

#### Leader Demographic Variables

To account for leader demographic characteristics, data on leader age, race, gender, education level, leader tenure, and leadership experience were collected. Age was treated as a continuous variable. Leader gender, race, and education level were treated as categorical variables. Education level is the highest degree attained, and categorized as Doctoral (DDS, DO, DrPH, DVM, JD, MD, PhD, or other doctoral degrees), or Master (MPH, MSN, MBA, or other master’s degree) or Bachelor (BA, BA, BSN, other bachelor degrees), or Associate degree (AD, ASN, other associate degrees). Leader tenure is the self-reported time that respondents have been in their current leadership positions and leadership experience relates to prior executive experience in other LHDs or organizations.

#### LHD Characteristics

As discussed in the theoretical framework, the agency characteristics of governance structure, and presence or absence of a board of health, medical director, and reserve fund were accounted for in the analysis. The change in LHD population size for 2012–2013 of the overall LHD revenue (multiple resources make up LHD revenues) and expenditure per capita for 2012–2013 were included in the analysis since it is anticipated that these elements influence the leader’s openness to change (40, 41).

### Analytical methods

The openness to change score is measured on a Likert scale, resulting in the ACQ score being treated as ordinal data with non-parametric tests used for examining the relationships between leader demographics and LHD characteristics. The Wilcoxon–Mann–Whitney non-parametric test, an analog to the independent samples *t*-test, was utilized to examine the differences in distribution of ACQ score by the leader’s gender, race, leadership experience, and the LHD attributes of a board of health, presence of a roll-over reserve fund, and presence of a separate medical director in the LHD. The Kruskal–Wallis test, the non-parametric analog to the ANOVA test, was performed on the leader’s highest degree obtained variable and LHD governance structure variable, both of which were treated as ordinal variables with more than two levels. To adjust for relevant potential confounders, linear regression analysis was performed.

## Results

### Leader and LHD characteristics

The results in leader characteristics (Table 1) demonstrated that the mean age of LHD leaders was 51 years and the average tenure as LHD top executive in current position is 6 years. The 2012–2013 average change in population size was approximately 260 persons with a range of approximately 1,600 persons exiting to about 4,450 persons entering a county (Table 2). Although an increase in population size for 2012–2013 was observed, the average 2012–2013 expenditure is approximately 6% less than the 2011–2012 average.

**Table 1:** **Leader and local health department characteristics**.

Variable	Descriptive statistics			
	Mean	SD	Min	Max
Leader age	51	9.39	30.49	73.72

Leader tenure	6	5.90	0.15	21.75

2012–2013 population change	260	1050	−1662	4456

2011–2012 revenue/capita	179.54	241.98	2.78	1136.99

2012–2013 revenue/capita	100.12	64.31	36.96	312.73

2011–2012 expenditure/capita	99.52	66.75	7.44	363.39

2012–2013 expenditure/capita	95.75	62.44	34.85	305.97

*2012–2013 population change = population of 2013 − population of 2012*.

**Table 2:** **Leader and local health department characteristics**.

Variable	Frequency	Relative frequency
**Gender**
Male	17	36.17
Female	29	61.70
Unknown	1	2.13

**Race**
White	42	89.36
African American	2	4.26
Others	2	4.26
Unknown	1	2.13

**Highest degree obtained**
Doctoral	8	17.02
Master’s	21	44.68
Bachelors	13	27.66
Associate degrees	2	4.26
Unknown	3	6.38

**Leadership experience**
First timers	40	85.11
Experienced	7	14.89

**Governance structure**
State government	2	4.26
Local government	11	23.40
Both state and local	32	68.09
Unknown	2	4.26

**Board of health**
Yes, present	44	93.62
No, absent	2	4.26
Unknown	1	2.13

**Separate medical director**
Yes	25	53.19
No	22	46.81

**Roll over reserve fund**
Yes, present	37	78.72
No, absent	6	12.77
Do not know	3	6.38
Unknown	1	2.13

Nearly 62% of leaders were females and approximately 36% were males (Table 2). The leadership racial profile demonstrated that over 89% were Caucasian, 4% African American, and 7% of other races. Approximately 45% of the leaders possessed a master’s degree, 28% held bachelor’s degrees, 17% a doctoral degree, and the other 10% held and associates or other degree. Over 85% of respondents were first time leaders while approximately 15% had previous leadership experience with other organizations or LHDs. As one of the few states practicing the shared governance model, 68% of Kentucky’s LHDs have both state and local governance, 23% have local governance, and about 4% have a state governance structure. More than 90% of all LHDs have a board of health. Over 78% of all LHDs have a reserve roll over funds and approximately 53% of the LHDs have a separate medical director.

### Relationship between instrument elements

To test for internal consistency and assess the strength of the relationship between the five items on the ACQ instrument, the Cronbach Alpha and Pearson Correlation tests were utilized. The Cronbach’s alpha of 0.77 is higher than the acceptable value of 0.70 as suggested by Nunnally and Bernstein (42) and implies that the internal consistency of the instrument is reliable. The moderate but statistically significant correlations between the five items (Table 3) suggest that there are no large collinearity concerns between the items on the instrument.

**Table 3:** **Openness to change intercorrelation matrix**.

Intercorrelation matrix				
Q1	Q2	Q3	Q4	Q5
–				

0.54***	–			

0.53***	0.29*	–		

0.42**	0.37**	0.39**	–	

0.36**	0.54***	0.24*	0.41**	–

*p < 0.05; **p < 0.01; ***p < 0.001.

### Variation in the leader’s openness to change

Few leaders strongly disagreed with any of the items on the ACQ instrument. Approximately 2% strongly disagreed that change is refreshing and that the organization becomes a deadening weight over time (Table 4). The findings suggest that an overwhelming majority were in agreement with the items on the ACQ instrument. Specifically, over 87% were in agreement (Strongly Agree or Agree) that there is something refreshing about enthusiasm for change, 81% agreed that leaders should be willing to devote more time to change activities, and 95% were in agreement that the current environment warrants an immediate response. Over 88% were in agreement that change must occur not only at an individual level but also at a system level and over 66% were in agreement that any organizational structure becomes a deadening weight over time and needs to be revitalized. For this final question with the lowest rate of agreement – two-thirds – one quarter of responders were neutral.

**Table 4:** **Variation in openness to change**.

Variable	Frequency distribution				
	Strongly disagree (%)	Disagree (%)	Neutral (%)	Agree (%)	Strongly agree (%)
Q1			12.77	51.06	36.17

Q2	2.13		17.02	57.45	23.40

Q3		2.13	2.13	72.34	23.40

Q4		2.13	10.64	42.55	44.68

Q5	2.13	6.38	25.53	44.68	21.28

*p < 0.05; **p < 0.01; ***p < 0.001.

*Q1. There is really something refreshing about enthusiasm for change; Q2. If I were to follow my deep convictions, I would devote more time to change movements. This seems to me to be a primary need today; Q3. The current situation in the community calls for change, we should do something now (we must respond at once); Q4. If you want to get anywhere, it is the policy of the system as a whole that needs to be changed, not just the behavior of isolated individuals; Q5. Any organizational structure becomes a deadening weight in time and needs to be revitalized*.

### Associations between ACQ score by leader and LHD attributes

Statistically significant correlations were observed between leader ACQ score, LHD, and leader characteristics (Table 5). The negative correlation between leader age and preceding year (2011–2012) revenues may be the determinant of intentions that shape the leader’s beliefs about the likely consequences of trying innovative strategies as discussed in the Section “Theory of Planned Behavior.” This could also reflect the adaptation element of the Flexible Leadership theory, whereby preference for earlier strategies that have worked (43) override the willingness to be open to change or may be the proxy measure of the leader’s educational level (44). The strong correlations between 2012–2013 revenues and expenditures were expected since revenues are integral to the delivery of programs and services (45), which subsequently increase the expenditures of the LHD. The moderately strong, statistically significant, correlations between the leader ACQ score and 2012–2013 revenues, and expenditures are of interest to this study as it suggests an underlying relationship between LHD characteristics and leader openness to change. The positive estimates imply that as the revenues and expenditures increase, leaders may be more willing to try innovative strategies. This reflects the management concept discussed in the Upper Echelon Theory whereby the financial health of the LHD informs leaders’ understanding of the situation and subsequently influences their openness to change.

**Table 5:** **Spearman correlation matrix for ACQ score, leader and LHD characteristics**.

Variable	Spearman correlations							
	1	2	3	4	5	6	7	8
1. Leader age	–							

2. Leader tenure	0.19	–						

3. 2012–2013 population change	−0.03	0.11	–					

4. 2011–2012 revenues/capita	−0.31*	−0.09	−0.47***	–				

5. 2012–2013 revenues/capita	0.12	0.14	−0.31*	0.19	–			

6. 2011–2012 expenditures/capita	0.02	0.11	−0.23	0.24	0.93***	–		

7. 2012–2013 expenditures/capita	0.12	0.13	−0.30*	0.18	0.99***	0.91***	–	

8. Openness to change score	0.17	0.09	0.18	−0.33*	−0.08	−0.16	−0.09	

*p < 0.05; **p < 0.01; ***p < 0.001.

### Variation in ACQ score by leader and LHD characteristics

The Wilcoxon–Mann–Whitney and Kruskal–Wallis tests provide evidence of statistically significant differences in rank average ACQ (r.a.ACQ) score by leader and LHD characteristics (Table 6). The r.a.ACQ score of the male leaders is 18.41 and that of female leaders is 26.48, indicating that female leaders have higher r.a.ACQ scores than male leaders. The significant differences in r.a.ACQ between male and female leaders may be attributed to the differences in leadership style between males and females (46). Studies have identified that women leaders prefer a participatory leadership style, such as transformational leadership, which correlates to female values developed through socialization processes that include building relationships, communication, consensus building, power as influence, and working together for a common purpose (47), all of which are integral for the management of a non-profit, governmental agency, such as an LHD. The significant differences in male and female leaders may also be a proxy measure representing the high number of LHD leaders that are woman and hold a nursing degree (48). In regard to racial diversity, the sample size is too small to make statistically significant conclusions concerning the observed differences in distribution of r.a.ACQ score. The Kruskal–Wallis test for differences in ACQ scores for leaders by the highest degree attained was statistically significant. Those with doctoral degrees had r.a.ACQ = 29; master’s degree holders r.a.ACQ = 25; associate and other degree holders r.a.ACQ = 24 and bachelor degree holders r.a.ACQ = 13. The leader educational level findings suggest that there are significant differences in the ACQ score based on the level of highest degree obtained by the leader, which corresponds to other study findings that identified important underlying relationships between the levels of education and leader openness to change (49).

**Table 6:** **Variation in ACQ score by leader and LHD characteristics**.

Variable	Mean ranks	*p*
**Gender**
Male	18.41	*
Female	26.48	

**Race**
White	21.92	*
African American	44.00	
Others	36.00	

**Highest degree obtained**
Doctoral	29.31	**
Master’s	25.45	
Bachelors	13.31	
Associate Degrees	24.00	

**Leadership experience**
First timers	22.75	
Experienced	31.14	

**Governance structure**
State government	28.5	
Local government	25.77	
Both state and local	21.70	

**Board of health**
Yes, present	23.38	
No, absent	26.00	

**Separate medical director**
Yes	22.26	
No	25.97	

**Roll over reserve fund**
Yes, present	24.35	
No, absent	20.00	

**N* = 47; continuity correction included; Mann–Whitney *U*-tests for gender, race, leadership experience, board of health, roll over reserve fund, and having a separate medical director under Ho: there is no difference in ACQ between samples; Kruskal–Wallis ANOVA test for highest degree obtained and governance structure under Ho: there is no difference in ACQ between samples. All numbers rounded to two decimal places*.

*p < 0.05; **p < 0.01; ***p < 0.001.

### Regression analysis

A backward elimination stepwise regression was utilized to develop a parsimonious model relating leader and LHD characteristics to openness to change. Of the financial metrics, only 2011–2012 revenue was included as 2012–2013 revenue, as well as 2011–2012 and 2012–2013 expenditures were highly collinear. Race also was not included due to lack of variability. Estimates of the final model after adjusting for confounders show that previous year LHD revenue (2011–2012) and educational level of the leader remain significantly associated with ACQ (see Table 7). The estimates reflect an interesting association between leader education level and openness to change. Relative to a doctoral degree, having a Bachelor’s or Associate degree results in leaders being less open to change. The same is not true for those with a Master’s level education implying that there is little difference in openness to change for those with any level of graduate education.

**Table 7:** **Leader and LHD predictors of ACQ**.

Variable	Estimate	*p*
Revenue/capita 2011–2012	−0.003	*

Gender	−0.89	
Ref: female		

Highest degree obtained		
Associate degrees	−1.74	
Bachelors	−2.63	*
Master’s	−0.36	
Ref: doctoral		

*p < 0.05; **p < 0.01; ***p < 0.001.

## Discussion

The study objectives are informed by the findings of the study. After adjustment for relevant potential confounders, both previous year revenue (2011–2012) and educational level of the leader remain associated with ACQ; however, the effect of gender and current year (2012–2013) revenue and expenditure is no longer statistically significant. The leader and LHD characteristics identified in the analysis correspond to elements of the theoretical framework, such as determinants of intention from the Theory of Planned Behavior, the adaptation element for the Flexible Leadership Theory, and managerial discretion from the Upper Echelon Theory, thus characterizing the nature of interaction between the leader, LHD characteristics, and openness to change. However, several limitations need to be acknowledged, the foremost being the inherent limitation of using a Likert scale self-assessment instrument that only establishes rank order and not the magnitude of openness to change. The small sample size (*n* = 47) limits the application of advanced analytics. The bivariate analyses that are sufficient for meeting the objectives of this study do not inform causal relationships between leader openness to change or leader and LHD characteristics. Also during the time in which the survey was fielded, state public health leaders were strongly encouraging LHD directors to consider operational changes, which may have influenced the leader ACQ score. Since in the analysis, only the 2011–2012 and 2012–2013 revenue, expenditures and population change were studied, this time period may be insufficient for observing the effects of leader and LHD characteristics on openness to change. Future studies will benefit from using a longitudinal study design that involves examining causal relationships between individual leader and LHD openness to change attributes.

## Conclusion and Implications

The findings of this study are similar to other openness to change studies (41, 50). In the current context of constant change and ongoing organizational turmoil, the findings are of particular interest to public health workforce development programs that are tasked with preparing leaders to deal with the complex and changing demands of critical public health services (51). Public health workforce development programs would benefit from creating opportunities that emphasize training leaders to recognize the complex interactions between individual leader and agency characteristics based on their openness to change and its subsequent impact on the ability to meet internal and external challenges (52, 53), such as the decline in the number of services offered by LHDs during the 2008–2010 recession (54) and the developing emphasis on emerging infections and deadly pathogens (55). In the interest of the wider public health field, these findings contribute to the literature on leadership attributes and organizational performance (56) that define leadership as a function of the interaction between the social situation and observable demographic characteristics of the leader (57) and the relationship between individual leader’s openness to change and agency performance (58).

## Conflict of Interest Statement

The authors declare that the research was conducted in the absence of any commercial or financial relationships that could be construed as a potential conflict of interest. The Associate Editor, Erik L. Carlton, declares that despite having collaborated with author James W. Holsinger Jr, the review process was handled objectively and no conflict of interest exists.
